# Feasibility of large-scale population testing for SARS-CoV-2 detection by self-testing at home

**DOI:** 10.1038/s41598-021-89236-x

**Published:** 2021-05-10

**Authors:** Paula Iruzubieta, Tatiana Fernández-Lanas, Laura Rasines, Lorena Cayon, Ana Álvarez-Cancelo, Alvaro Santos-Laso, Agustín García-Blanco, Soraya Curiel-Olmo, Joaquín Cabezas, Reinhard Wallmann, Emilio Fábrega, Víctor M. Martínez-Taboada, José L. Hernández, Marcos López-Hoyos, Jeffrey V. Lazarus, Javier Crespo

**Affiliations:** 1 Gastroenterology and Hepatology Department, Marqués de Valdecilla University Hospital, Clinical and Translational Digestive Research Group, University of Cantabria, IDIVAL, Santander, Spain; 2 Division of Epidemiology and Computational Biology, Cantabria University School of Medicine, Santander, Spain; 3 Division of Rheumatology, Marqués de Valdecilla University Hospital, IDIVAL, Santander, Spain; 4 Department of Internal Medicine, Marqués de Valdecilla University Hospital, IDIVAL, University of Cantabria, Santander, Spain; 5 Immunology Department, Marqués de Valdecilla University Hospital, IDIVAL, Santander, Spain; 6 Barcelona Institute for Global Health (ISGlobal), Hospital Clínic, University of Barcelona, Barcelona, Spain

**Keywords:** Diseases, Health care

## Abstract

The simplicity and low cost of rapid point-of-care tests greatly facilitate large-scale population testing, which can contribute to controlling the spread of the COVID-19 virus. We evaluated the applicability of a self-testing strategy for SARS-CoV2 in a population-based, cross-sectional study in Cantabria, Spain, between April and May 2020. For the self-testing strategy, participants received the necessary material for the self-collection of blood and performance of a rapid antibody test using lateral flow immunoassay at home without the supervision of healthcare personnel. A total of 1,022 participants were enrolled. Most participants correctly performed the COVID-19 self-test the first time (91.3% [95% CI 89.4–92.9]). Only a minority of the participants (0.7%) needed the help of healthcare personnel, while 6.9% required a second kit delivery, for a total valid test result in 96.9% of the participants. Incorrect use of the self-test was not associated with the educational level, age over 65, or housing area. Prevalence of IgG antibodies against SARS-CoV2 for subjects with a valid rapid test result was 3.1% (95% CI 2.2–4.4), similar to the seroprevalence result obtained using a conventional approach carried out by healthcare professionals. In conclusion, COVID-19 self-testing should be considered as a screening tool.

## Background

The high transmissibility of SARS-CoV2, even in asymptomatic patients, indicates that diagnosis based on symptoms and contact tracing alone is insufficient to contain the COVID-19 pandemic. Moreover, restrictions on population movement, closure of certain businesses and activities, and full or partial lockdowns can cause serious socioeconomic consequences for any country^1^. For this reason, mass population testing is necessary before effective vaccines or antiviral drugs are available^2^.

Detection of viral genome by reverse transcription polymerase chain reaction (RT-PCR) performed with respiratory specimens, especially with nasopharyngeal swabs, are the cornerstone of SARS-CoV2 infection diagnostic testing^3^. These techniques require specialized healthcare personnel, centralized laboratory facilities, and time to provide results; therefore, the widespread use of these techniques has economic and logistical limitations.

Recently, point-of-care rapid tests have been developed with a high diagnostic accuracy^4–7^. Rapid antibody tests, using capillary blood, can identify ongoing, recovering, or previous SARS-CoV2 infection, which is important to develop vaccination programs since vaccine doses could be saved in seropositive subjects at a time when the speed of vaccination is slow and vaccine availability limited^8,9^. Rapid antigen tests detect the presence of viral proteins expressed by SARS-CoV2 in samples from nasopharyngeal swabs identifying subjects with an acute infection^7^. Moreover, recent studies showed that saliva is useful in diagnosing COVID-19^10^. This has the advantage of being easily self-collected by the subject. The simplicity and low cost of these rapid tests greatly facilitate the logistics of mass population testing. Telemedicine could further optimize these techniques through avoiding visiting healthcare facilities and, therefore, the risk of contact with potentially contagious subjects, meaning that control of the pandemic spread could be possible. Hence, the implementation of public health strategies focused on COVID-19 self-testing and telemedicine should be a priority for governments worldwide. However, experience of unsupervised home self-testing for SARS-CoV2 detection in the general population with rapid tests is not well studied. Therefore, we designed a self-testing strategy for SARS-CoV2 detection in a representative sample of the general population in order to evaluate its applicability as well as feasibility and acceptability by the community.

## Methods

### Study population and design

**Figure 1:**
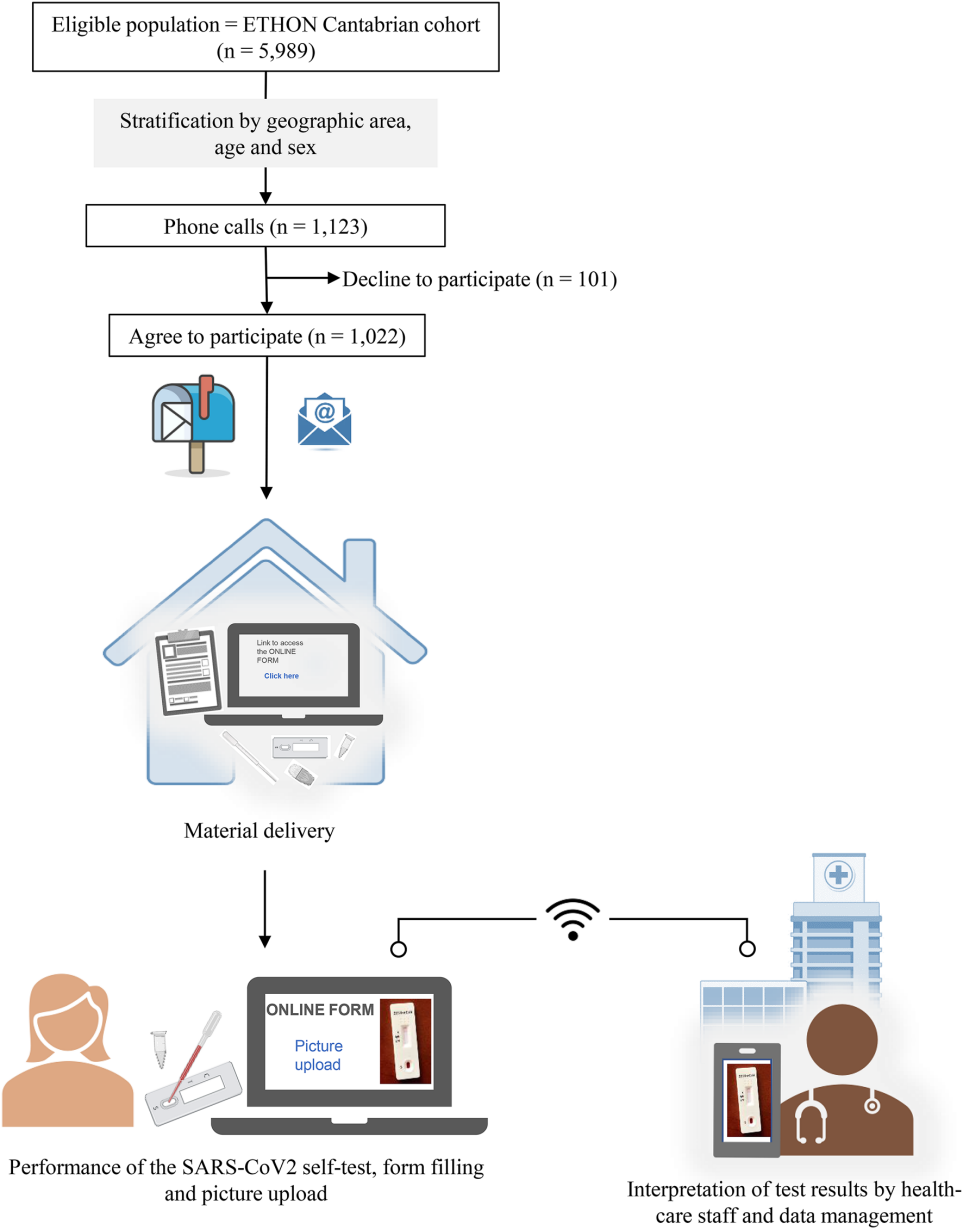
Study Flow chart and summary of its methodology. Created with support of BioRender (https://biorender.com/), 2021.

A population-based, cross-sectional study nested in the ETHON (EsTudio poblacional de enfermedades Hepáticas naciONal) cohort^11^ was designed. The ETHON cohort compromises of 5,989 inhabitants from the region of Cantabria in northern Spain. Participants were selected through a random and representative sample by means of sampling by two-stage conglomerates with stratification according to economic status, housing area (rural/urban), and age, being representative of the general population. Personal data, such as date of birth, nationality, gender, and level of education, were available for all subjects. From this cohort, we selected 1,123 subjects using stratified sampling according to geographic area, age, and gender. We assumed a prevalence of SARS-CoV-2 infection between 4–8% and considered the population of the Community of Cantabria (584,000 inhabitants), a confidence level of 95%, a margin of error of 2%, and estimated a loss of 30% of the included subjects. Selected subjects were contacted by telephone and were invited to participate. The Ethics Committee of investigation of Cantabria approved this study in 2020 (code 2020.176) and written informed consent was obtained from all participants. All research was performed in accordance with the Declaration of Helsinki. The flow chart and design of the study are detailed in Fig. 1.

### Material delivery and data collection

All subjects who agreed to participate received, by conventional mail, an envelope containing: a) a bag with the rapid antibody test, one micropipette, two lancets, and two Eppendorf tubes with approximately 50ul of buffer each; b) paper-based instructions for use with an available telephone number to request help; and c) informed consent and pre-stamped envelope for subsequent forwarding once signed. A link to a web-based form was sent to the participants by email or SMS text message.

The design of the web-based form aimed to collect relevant information on the socio-demographic characteristics, medical history, and symptoms and sources of SARS-CoV-2 infection. A video tutorial was recorded to show how to perform the SARS-CoV-2 antibody rapid test and it was embedded in the form with a space for uploading the pictures with the test result. At the end of the process, participants could complete a satisfaction survey. The form was created as an online survey with Typeform (Typeform S.L.; Barcelona, Spain; https://www.typeform.com). A full copy of the form as well as the paper-based and video-based instructions for use are available as supplementary material (see Appendix S1, Appendix S2 and Supplementary video).

The recruitment of participants and material delivery was conducted between April 27 and May 29, 2020. The test results, shown in the pictures uploaded by participants, were interpreted almost immediately by healthcare personnel. All individuals with a negative serological result were notified by email or SMS text message, and those with positive serological result were notified by a phone call.

### SARS-CoV2 antibody rapid tests

The commercial kit Lungene COVID-19 IgG/IgM Rapid Test Cassette (Hangzhou Clongene Biotech Co., Ltd., China) was used to detect both IgG and IgM antibodies. This test uses colloidal gold immunochromatography in point-of-care format on blood, serum, or plasma samples and offers the result in 15 min. The manufacturer reported sensitivity of 97.4% for IgG and 87.01% for IgM and specificity of 98.89% for both IgG and IgM, using RT-PCR as the gold standard. Due to the lower sensitivity and shorter duration of IgM, results for the rapid test reported here are based only on IgG.

### Feasibility and applicability of the self-testing strategy

The feasibility was defined as participant’s ability to complete the self-test obtaining a valid test result without healthcare personnel support (telephone or in person), and to correctly use the different kit components in a home-based, unsupervised setting. We considered an incorrect use of the self-test when sending a second kit was necessary, the control band did not appear in the rapid test picture sent, and specialized healthcare personnel assisted in its performance. A satisfaction survey, concerning the instructions included, the picture uploading process, the handling of the lancet, the pipette and the rapid antibody test, was used to evaluate the difficulty levels encountered by the participants. The opinion about testing at home and the need for healthcare personnel to perform this type of tests were evaluated as well.

The applicability of our strategy was considered if the seroprevalence result obtained was equivalent to the seroprevalence result obtained using a conventional approach, ie tests carried out by healthcare professionals.

### Statistical analysis

Seroprevalence was estimated as the proportion of individuals who had a positive result in the IgG band. Qualitative variables were expressed as absolute values and proportions and quantitative variables as means and standard deviations. Missing values were ignored. Categorical variables were compared with the chi-square test or Fisher’s exact test as appropriate. Normally distributed values were analyzed by Student’s t-test, and those non-normally distributed were assessed by Mann–Whitney U-test. The Wilson score interval was used to estimate the 95% confidence intervals. Statistical analyses were performed using SPSS Statistics version 21.0 (IBM Corporation, USA).

## Results

### Study population

Of 1,123 selected subjects, 101 (9%) declined to participate (Fig. 1). The 1,022 study participants received the rapid test kit at their homes and the online form. Demographic characteristics and medical history of participants are shown in Table 1. Overall, the mean age was 51.5 years (range 18 to 83), and 531 (52%) were female. The proportion of subjects with obesity (21.4%) and diabetes (7%) was consistent with the reported prevalence of these comorbidities both in Spain and in Europe^12–14^. Around one half of participants reported symptoms suggestive of COVID-19 in the past three months, but only 79 (7.7%) participants were previously subjected to molecular testing to detect SARS-CoV2 infection, of whom four (5%) had a positive result.

**Table 1:** Demographic characteristics and medical history of the study participants.

	Total	IgG antibody negative subjects	IgG antibody positive subjects	*P*
	(n = 1022)	(n = 959)	(n = 31)	
**Age**				
18–49	468 (45.8)	449 (46.8)	9 (29)	ns
50–64	274 (26.8)	252 (26.3)	9 (29)	
≥ 65	280 (27.4)	258 (26.9)	13 (41.9)	
Gender, F/M	531 (52) / 491 (48)	491 (51.2) / 468 (48.8)	19 (61.3) / 12 (38.7)	ns
Housing area, rural/urban	241 (24.3) / 749 (75.7)	234 (24.4) / 725 (75.6)	7 (22.6) / 24 (77.4)	ns
Obesity	218 (21.4)	209 (21.9)	5 (16.1)	ns
Arterial hypertension	245 (24)	227 (23.7)	9 (29)	ns
Diabetes	72 (7)	69 (7.2)	1 (3.2)	ns
Chronic lung disease	44 (4.3)	40 (4.2)	2 (6.5)	ns
Cardiac pathology	54 (5.3)	52 (5.4)	1 (3.2)	ns
Immunosuppressive treatment	47 (4.6)	42 (4.4)	3 (9.7)	ns
Cancer diagnosis in the last 5 years	40 (3.9)	37 (3.9)	2 (6.5)	ns
COVID-19 symptoms in the last 3 months	515 (50.4)	475 (49.5)	25 (80.6)	< 0.01
Previous negative SARS-CoV2 PCR test	75 (7.3)	70 (7.3)	4 (12.9)	ns
Previous diagnosis of COVID-19 by PCR	4 (0.4)	1 (0.1)	2 (6.5)	< 0.01
**Rapid test results**				
Positive IgM	11 (1.1)	11 (1.1)	–	
Positive IgG	28 (2.7)	-	28 (90.3)	
Positive IgM + IgG	3 (0.3)	-	3 (9.7)	-
Absence of control band	32 (3.1)	-	-	

Qualitative variables are expressed as absolute values and proportions. Comparisons between groups of IgG antibody negative subjects and IgG antibody positive subjects were performed with Fisher's exact test or Pearson's chi-square test as appropriate.

### Difficulties of the population for self-testing of SARS-CoV2 detection

**Figure 2:**
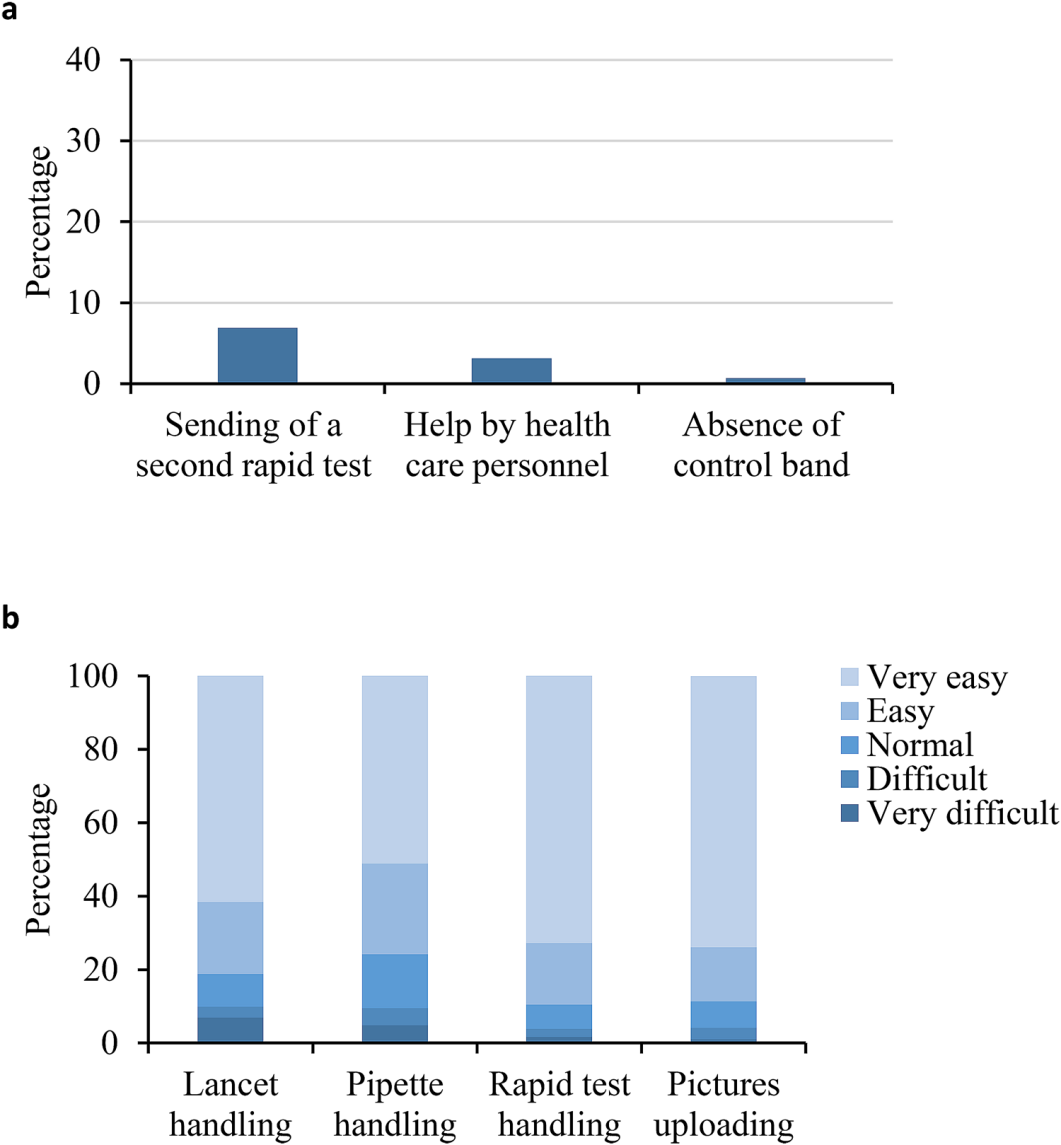
Difficulties of the population for self-testing of SARS-CoV2 detection. (**a**) Percentage of participants with an incorrect use of self-test. (**b**) Degree of difficulties reported by participants regarding the use of the different kit components and perform the COVID-19 self-test.

Most of participants (96.9% [95% CI 95.6–97.8]) obtained a valid test result, with 96.2% (95% CI 94.8–97.2) of participants able to correctly perform the self-testing procedure without healthcare support. The feasibility of the self-test was also assessed by determining the participants who required a second kit delivery (71, 6.9%), help from healthcare personnel (7, 0.7%), and the absence of test control band in the uploaded pictures (32, 3.1%) (Fig. 2a). No control band was observed in 21 of the 71 participants (29.6%) who received a second kit. However, most of participants correctly performed the COVID-19 self-test the first time (91.3% [95% CI 89.4–92.9]). The mean age of subjects with an incorrect use of the self-test was slightly higher, but statistically significant, compared to subjects with a correct use of the self-test (54.4 [SD 13.2] vs 51.2 [SD 14.6]; *P* = 0.038). No differences were found regarding educational level, age over 65 years, and housing area between subjects who performed self-test correctly and those who did not (Table 2).

**Table 2:** Characteristics of the study participants according to the use of the self-test.

	Correct use of the self-test (n = 933)	Incorrect use of the self-test (n = 89)	*P*
**Age**			
18—49	440 (47.2)	28 (31.5)	
50–64	238 (25.5)	36 (40.4)	0.004
≥ 65	255 (27.3)	25 (28.1)	
**Housing area**			
Rural	219 (23.5)	28 (31.5)	ns
Urban	714 (76.5)	61 (68.5)	
**Educational level**			
Primary education	97 (14)	16 (18)	
Secondary education	507 (54.3)	46 (51.7)	ns
Post-secondary education	198 (21.2)	23 (25.8)	
No answer	131 (14)	4 (4.5)	

Qualitative variables are expressed as absolute values and proportions. Comparisons between groups of subjects with a correct use of the self-test and subjects with an incorrect use of the self-test were performed with Fisher's exact test or Pearson's chi-square test as appropriate.

The degree of difficulties and satisfaction reported by participants regarding the entire process and performance of the COVID-19 self-test are shown in Fig. 2b and Table 3. Overall, the handling of the lancet, pipette, and rapid test was easy or very easy for 81.2% (95% CI 77.5–84.4), 75.8% (95% CI 71.8–79.3) and 89.6% (95% CI 87.6–91.3) of participants, respectively. Similarly, most participants (88.6% [95% CI 85.5–91.1]) considered the uploading of pictures easy or very easy. More than 90% of the respondents were satisfied or very satisfied with the information received and had no need to leave home. Slightly more than a third (38.2%) of the respondents believed it necessary to carry out this type of test by healthcare personnel.

**Table 3:** Results of the satisfaction survey questions.

	N (%)*
**The information received to access and complete the survey has been adequate**	
Very dissatisfied	0 (0)
Dissatisfied	12 (1.2)
Neutral	36 (3.6)
Satisfied	170 (17.2)
Very satisfied	772 (78)
**The information received to perform the coronavirus test has been adequate**	
Very dissatisfied	4 (0.4)
Dissatisfied	8 (0.8)
Neutral	56 (5.6)
Satisfied	156 (15.7)
Very satisfied	772 (77.5)
**The video tutorial has been useful**	
Very dissatisfied	6 (0.6)
Dissatisfied	10 (1)
Neutral	36 (3.6)
Satisfied	132 (13.3)
Very satisfied	808 (81.5)
**Access to the form has been easy**	
Very dissatisfied	4 (0.4)
Dissatisfied	24 (2.4)
Neutral	56 (5.7)
Satisfied	168 (17)
Very satisfied	738 (74.5)
**The form format is simple**	
Very dissatisfied	2 (0.2)
Dissatisfied	22 (2.2)
Neutral	48 (4.9)
Satisfied	154 (15.7)
Very satisfied	756 (77)
**The questions have been easy to understand**	
Very dissatisfied	2 (0.2)
Dissatisfied	4 (0.4)
Neutral	24 (2.4)
Satisfied	150 (15.1)
Very satisfied	812 (81.9)
**Uploading picture has been:**	
Very difficult	10 (1)
Difficult	32 (3.3)
Normal	70 (7.1)
Easy	146 (14.8)
Very easy	726 (73.8)
**The handling of the lancet has been:**	
Very difficult	68 (6.9)
Difficult	30 (3)
Normal	88 (8.9)
Easy	194 (19.6)
Very easy	610 (61.6)
**The handling of the pipette has been:**	
Very difficult	48 (4.9)
Difficult	46 (4.7)
Normal	146 (14.7)
Easy	244 (24.6)
Very easy	506 (51.1)
**In general, the handling of the rapid test has been:**	
Very difficult	17 (1.7)
Difficult	22 (2.1)
Normal	67 (6.6)
Easy	170 (16.7)
Very easy	742 (72.9)
**I really appreciate, in this pandemic situation, for this test-performing system at home**	
Very dissatisfied	14 (1.4)
Dissatisfied	14 (1.4)
Neutral	40 (4)
Satisfied	122 (12.3)
Very satisfied	806 (80.9)
**Do you think that this king of test should be carried out by healthcare personnel?**	
Not necessary	538 (54)
Yes, always	380 (38.2)
Don’t know	78 (7.8)

*Percentages are calculated from non-missing values.

### Applicability of the mass self-testing strategy

To assess the applicability of our strategy, we compared our seroprevalence results with the results obtained in the nationwide population-based seroepidemiological study in Spain^15^, which was carried out between April 27 to June 22, 2020, overlapping with our study.

The prevalence of IgG antibodies against SARS-CoV2 for subjects with a valid rapid test result was 3.1% (95% CI 2.2–4.4). Demographic characteristics and medical history according to the positivity for IgG of participants are shown in Table 1. Seroprevalence was higher in participants aged 50 years or older (4,2% [95% CI 2.8–6.3]) compared to other adults (1.9% [95% CI 1–3.6]) (*P* = 0.04). It is noteworthy that asymptomatic cases represented 19.4% of all IgG-positive participants, percentage similar to that reported by the ENE-COVID study^16^. Estimated seroprevalence for the Cantabrian region by the point-of-care test in that study was 3.6% (95% CI 2.3–5.7), finding no significant differences with the seroprevalence found in our study (*P* = 0.54).

## Discussion

We evaluated an extensive self-testing strategy carried out in northern Spain with representative individuals of the general population, and demonstrate a high level of feasibility and applicability of SARS-CoV2 serological screening with a rapid antibody self-test. The study was carried out in a population previously untrained in health-related digital technologies, and we analyzed the effectiveness and potential barriers to the implementation of this intervention. Over 95% of the participants were able to correctly perform the self-testing procedure without assistance from healthcare personnel. Incorrect use of the self-test was more frequent among subjects aged 50 to 64 years but not in those older than 65 years. However, this incorrect use was not associated with the education level. These results show that a low educational level does not challenge understanding the instruction for use or in handling the different components of the rapid antibody tests, possibly due to the support provided by the video tutorial.

The COVID-19 pandemic has been successfully controlled in some Asian-Pacific countries. This success is largely due to extensive testing, contact tracing, and isolating of all cases from the beginning of the outbreak, which have each been reinforced by innovative surveillance technology^17–19^. This demonstrates that testing, and in particular, mass testing, is crucial^20^. In fact, the World Health Organization highlighted the importance of testing for COVID-19 surveillance to limit the spread of the disease, enable public health authorities to manage its risk, and thereby restore normal economic and social activities^21^. However, as it has been demonstrated in different Western countries, one of the most pressing logistical difficulties in controlling the pandemic is conducting mass testing. Unlike Western countries, many Asian countries, with experience in major epidemics, have a robust and well-equipped infrastructure for medical care and public health to handle an infectious outbreak; and most of its population is better conditioned to cooperate with strict rules and invasive surveillance in times of crisis^22,23^. However, given the great advances in diagnostic tests for SARS-CoV-2 infection, no major logistical changes are required to carry out mass testing. For this reason, we believe that mass self-testing with the support of digital technologies for rapid reporting, data management, and analysis, can help identify infected subjects, whether or not they are symptomatic. This type of self-screening approach could also have a great impact on a smaller scale through targeting smaller populations with a high risk of contagion, such as nursing home, hospitals, and reception centers. Self-testing in these settings has been proven useful for surveillance of other infectious diseases such as HIV^24,25^.

To date, there are two published works that evaluated the usability of SARS-CoV-2 antibodies self-testing at home^26,27^. In France, Tonen-Wolyec et al. demonstrated high practicability of self-testing, but the performance of the self-test was supervised by an observer and the sample size was small (n = 167)^26^. In England, Atchison et al. selected at random and posted rapid antibody tests to 14,400 subjects evaluating the usability through an online survey^27^. Most participants (more than 90%) reported a valid result. However, their study admitted several participants per household which could influence the overall result about participant’s ability. Both studies reflect the potential use of COVID-19 self-testing for serological immune status assessment by the general public. The main advantage of our study resides in a large sample size that represents the general population given the randomization design for the participants selection, which reflects a more realistic scenario. Moreover, unlike Atchison C et al., we review all uploaded test pictures, and thus we used an objective measure to evaluate the feasibility of SARS-CoV-2 self-test in the general population.

Our study was carried out during the last months of the first wave of the COVID-19 pandemic, and it is noteworthy that, despite the abundance of information regarding COVID-19 in the media and the announcements of the Spanish government about the success in controlling the pandemic, only 9% of eligible subjects declined to participate. This reflects the existing concern and the willingness of the population to collaborate with innovative control measures. Over 80% of participants reported being very satisfied with the test-performing system at home. Most of participants considered that healthcare personnel were not necessary to carry out this type of test, but 38% of participants did, probably due to the need to obtain a blood sample for this rapid test. In this sense, the promising role of saliva in the diagnosis of SARS-CoV-2 infection could facilitate self-testing. It can also facilitate mass testing without the need for sanitary facilities and provide test results within a few minutes due to the recently developed rapid saliva tests^10^. However, SARS-CoV-2 self-testing does raise ethical concerns^28^. False negatives have the potential to cause harm, and false positives might produce psychological distress. Hence, effective linkage to care services is key to expand self-testing for SARS-CoV-2.

We do not only show that mass self-testing is possible but we also verified its applicability by comparing the results regarding seroprevalence obtained in our cohort with the prevalence rate reported by the ENE-COVID study for the Cantabria region^16^. This study showed seroprevalence of SARS-CoV-2 infection in Spain at national and regional level with more than 61,000 participants through point-of-care rapid test, performed by healthcare personnel. The results obtained in both studies for our region were practically identical (3.1% vs 3.6%). This result highlights the applicability of the SARS-CoV-2 surveillance strategy through self-testing. Although we did not conduct an economic evaluation of our self-testing strategy, we did not receive any sponsorship for the realization of this study, which shows the efficiency of this approach.

This study is subject to several limitations. Firstly, it may take up to five days after infection to detect SARS-CoV-2 antibodies^29^. However, when the study was designed, Spain did not offer another type of rapid test. Yet, surveillance of antibody seropositivity in a population can allow inferences to be made about the extent of infection and the cumulative incidence of infection in the population, and thus develop efficient vaccination programs. The main objective of our study was to demonstrate that self-testing is possible and it is independent of the type of test used. Furthermore, we think that if the general population is capable of performing a self-test for antibodies detection with a blood sample, they will be able to perform a self-test using saliva. However, specific studies are necessary to evaluate the oral fluid-based self-testing as a screening tool for SARS-CoV-2 infection in general population. Another potential limitation of the use of rapid tests could be their lower sensitivity compared to laboratory-based tests, hence the importance of linking the self-test users to formal healthcare services. Nevertheless, it is important to highlight that in this time of crisis, the speed of reporting cases is much more important than the sensitivity, since the delay in diagnosis to detect asymptomatic individuals compromises the surveillance program's effectiveness^30^. Moreover, several recent studies demonstrated an absence of contagiousness when the concentration of viral particles is fewer than 10^6^ copies per milliliter in samples collected from patients, a concentration that is already detected by the rapid antigen tests^31,32^. Another limitation of our study is self-reporting using an unsupervised, at-home self-test. Besides, we did not ask about assistance by cohabitants or family members because we consider it normal in the face of a new activity or situation. Finally, it is evident that the sole use of mass tests will not be enough to control the pandemic, but it will be beneficial as part of the package of control efforts. Before carrying out strategies of this type, an exhaustive information campaign in both public and private media and social networks will be required. Also required is the responsibility of every individual to perform the self-test, an adequate technological platform that informs in real-time both individual and health authorities simultaneously, the acceptance of confinement in the case of a positive result, even if you are asymptomatic, and the mobilization of all social resources to facilitate aforementioned confinement. However, these aspects were not part of the objectives of this study.

Our results from Spain lead us to conclude that COVID-19 self-testing in other high-income countries can be applied as a mass screening tool. If supported by digital technologies, it could constitute a key control measure to limit the spread of SARS-CoV-2 infection.

### Ethics approval

The Ethics Committee of investigation of Cantabria approved this study (code 2020.176) and written informed consent was obtained from all participants.

## Supplementary Information

Supplementary Information 1.

Supplementary Video 1.

## Data Availability

The datasets used and/or analysed during the current study are available from the corresponding author on reasonable request.
