# Supplementary material for: Feasibility of large-scale population testing for SARS-CoV-2 detection by self-testing at home
Source: Sci Rep. 2021 May 10;11:9819. doi: 10.1038/s41598-021-89236-x (PMC8110575; doi:10.1038/s41598-021-89236-x)
Supplement: Supplementary file 1 — Supplementary Information 1. [file 41598_2021_89236_MOESM1_ESM.pdf]

Feasibility of large-scale population testing for SARS-CoV-2 detection by self-testing at home.

Paula Iruzubieta, Tatiana Fernández-Lanas, Laura Rasines, Lorena Cayon, Ana Álvarez-Cancelo, Álvaro Santos-Laso, Agustín García-Blanco, Soraya Curiel-Olmo, Joaquín Cabezas, Reinhard Wallmann, Emilio Fábrega, Víctor M. Martínez-Taboada, José L. Hernández, Marcos López-Hoyos, Jeffrey V Lazarus, Javier Crespo.

Appendix S1: FULL COPY OF THE QUESTIONNAIRE

This questionnaire had an online format. Here we transcribe the questions.

SOCIODEMOGRAPHIC DATA

1. Kit number: _____________________

2. Current date: _______________________

3. Date of birth: _______________________

4. Gender

☐ Male	☐ Female

5. Zip code from your habitual residence: _______________

6. Home-related information:

- How many people live in your habitual residence (including you)?

☐ 1	☐ 2	☐ 3	☐ 4	☐ 5 or more

- Dwelling size (in square meter – m^2 –): _____________________
- Number of bedrooms: _______________
- Are there individuals who do not belong to the household who visit your habitual residence regularly?

  ☐ Yes, the domestic assistant

  ☐ Yes, the caregiver of a dependent person

  ☐ Yes, others

☐ No

7. In case of living with someone:

   a. Has any partner had respiratory symptoms (cough, shortness of breath or fever) in the last months?

      ☐ No

      ☐ Yes

   b. Has any partner been diagnosed with infection by the new coronavirus?

      ☐ No

      ☐ Yes

8. Do you work in any of these sectors?

   ☐ Shop assistant

   ☐ Transport service

   ☐ Security corps and forces, firefighter, civil protection

   ☐ Cleaning service

   ☐ Health personnel with clinical assistance to patients

   ☐ Social services personnel

   ☐ Caregiver of a dependent person at home

   ☐ Other face-to-face services during the pandemic period

9. Have you been in contact with someone who has been diagnosed with infection by the new coronavirus?

   ☐ No

   ☐ Yes

CLINICAL DATA

10. Weight (kg): __________________

11. Height (cm): ___________________

12. Are you a smoker or have been a smoker (more than 1 cigarette a day) in the last 5 years?

☐ Yes	☐ No

13. Do you take medication for the following health problems?

   ☐ High blood pressure

☐ Diabetes (high blood levels of glucose)

☐ Chronic lung disease (such as COPD or chronic bronchitis)

☐ Heart disease (such as heart attack or angina pectoris)

☐ Some disease requiring the continuous intake of medication that lowers your defenses (immunosuppressants) including corticosteroids, azathioprine, methotrexate, tacrolimus, infliximab, etc…

14. Have you been diagnosed with cancer in the last 5 years?

☐ Yes	☐ No

15. From February 1, 2020 to today: Have you had any of the following symptoms?

Symptom	Answer	
Fever	☐ Yes	☐ No
Cough	☐ Yes	☐ No
Fatigue or shortness of breath	☐ Yes	☐ No
Severe asthenia (you felt very tired)	☐ Yes	☐ No
Muscle or joint pain	☐ Yes	☐ No
Sore throat	☐ Yes	☐ No
Headache	☐ Yes	☐ No
Loss of smell	☐ Yes	☐ No
Disturbance of flavors perception	☐ Yes	☐ No
Nausea or vomiting	☐ Yes	☐ No
Diarrhea	☐ Yes	☐ No
Others:		

16. Have you ever been tested to detect if you were infected with coronavirus by taking a sample from your throat or nose?

☐ No
☐ Yes, with a negative result
☐ Yes, with a positive result

17. If your previous answer (question 16) is affirmative, please, specify the reason why it was done.

☐ Have symptoms of respiratory infection (cold or pneumonia)
☐ Contact with a person infected by coronavirus
☐ Be health personnel
☐ Others (specify)

18. If your previous answer (question 16) is affirmative, please, specify the approximate date you were tested for coronavirus infection.

19. If your previous answer (question 16) is affirmative:

a. A medical doctor prescribed you specific medication (not pain-relief treatment such as acetaminophen)?

☐ Yes

☐ No

b. Were you hospitalized?

☐ Yes

☐ No

c. Were you admitted to an Intensive Care Unit (ICU)?

☐ Yes

☐ No

SATISFACTION QUESTIONNAIRE

Please, indicate your degree of satisfaction with the following aspects regarding:

- **Information received:**

1. The information received to access and complete the survey has been adequate.
   ☐ Very dissatisfied
   ☐ Dissatisfied
   ☐ Neutral
   ☐ Satisfied
   ☐ Very satisfied

2. The information received to perform the coronavirus test has been adequate.
   ☐ Very dissatisfied
   ☐ Dissatisfied
   ☐ Neutral
   ☐ Satisfied
   ☐ Very satisfied

3. The video tutorial has been useful.
   ☐ Very dissatisfied
   ☐ Dissatisfied
   ☐ Neutral
   ☐ Satisfied
   ☐ Very satisfied

- **Online form:**

1. Access to the form has been easy.
   ☐ Very dissatisfied
   ☐ Dissatisfied
   ☐ Neutral
   ☐ Satisfied
   ☐ Very satisfied

2. The form format is simple.
   ☐ Very dissatisfied
   ☐ Dissatisfied

☐ Neutral
☐ Satisfied
☐ Very satisfied

3. The questions have been easy to understand.
   ☐ Very dissatisfied
   ☐ Dissatisfied
   ☐ Neutral
   ☐ Satisfied
   ☐ Very satisfied

4. Pictures uploading has been a procedure:
   ☐ Very difficult
   ☐ Difficult
   ☐ Normal
   ☐ Easy
   ☐ Very easy

- **<u>Rapid antibodies test:</u>**

1. The handling of the lancet (stinging device to obtain a small amount of blood) has been:
   ☐ Very difficult
   ☐ Difficult
   ☐ Normal
   ☐ Easy
   ☐ Very easy

2. The handling of the pipette (tube to transfer blood from finger to well) has been:
   ☐ Very difficult
   ☐ Difficult
   ☐ Normal
   ☐ Easy
   ☐ Very easy

3. In general, the handling of the rapid test has been:
   ☐ Very difficult
   ☐ Difficult
   ☐ Normal
   ☐ Easy
   ☐ Very easy

Your opinion is important, please answer the following aspects or questions:

- **I really appreciate, in this pandemic situation, for this test-performing system at home.**
  ☐ Very dissatisfied
  ☐ Dissatisfied
  ☐ Neutral
  ☐ Satisfied
  ☐ Very satisfied

- **Do you think that this kind of test should be carried out by health care personnel?**

  ☐ YES, always ☐ NO, it is not necessary ☐ I don´t know

- **What aspect/s of the study would you improve? ________________________________**

Appendix S2: FULL COPY OF THE PAPER-BASED INSTRUCTION FOR USE

<u>GUIDELINES</u>

**Record ID:**
**Kit number:**

Please, in this envelope you can find all the documents and materials required for participate in the study you already know, since you have agreed to participate in it by telephone. However, and in compliance with the corresponding code of ethics, we should kindly ask you to, please, sign the document *Informed Consent* and send it back to us by ordinary postal mail, as explained below. Herein, you will find all the information about the content of the envelope and the steps that you should follow for performing the test. **<u>Therefore, please, read this document carefully and in detail</u>**.

CONTENT OF THE ENVELOPE:

I. Documents:
   a) Patient Information Sheet
   b) Informed Consent (2 identical copies)

II. Materials for self-performing the antibodies test (see the images below):
   a) Lancet
   b) Test
   c) Pipette
   d) Liquid-containing tube (i.e., buffer)

STEPS TO FOLLOW:

I. <u>Documents</u>:

1º) You will find the *Patient Information Sheet* and two copies of the *Informed Consent*. In case of any of these documents are missing, please do not hesitate to contact us immediately (using the telephone number indicated at the end of this document). Please, read carefully and in detail the *Patient Information Sheet* that provides you information about the study.

2º) Fill in, at least, one of the *Informed Consent* copies (the another one is for you). Please, take into consideration the following indications to correctly fill in the *Informed Consent* document:

- In the first page, you should write your name and surname in CAPITAL LETTERS, as well as, you should answer the 4 questions, that you will find in the above mentioned document, choosing between the options “Yes” or “No”.
- In the second page, you should sign the document and write the current date in the space reserved for it (i.e., up to the right). **IMPORTANT:** At the moment, you **do not should** complete the last part of this document (i.e., withdrawal of consent); this part should be only completed later on if you want to withdraw your consent to participate in the study.

3º) Introduce the signed *Informed Consent* in the envelope containing the Valdecilla's Hospital address and deposit it in any mailbox or post office.

II. Materials for performing both the test and survey:

1º) Use an electronic device (i.e., computer, smartphone or tablet) to access the following URL, where you will find a survey and a video tutorial:

https://digestivovaldecilla.typeform.com/to/BKqrXL (this is the link that you should have received in your smartphone or email account)

Watch carefully the video that you will find at the beginning of the survey and which will explain you, in detail, the usefulness of each instrument and how to perform the test yourself.

2º) Please, follow these instructions: Wash your hands with hot water. Spin the green tip of the lancet and remove it. Place the lancet on your finger and tight. Use the pipette to suck an enough volume of blood (i.e., large drop of blood) and place it into the test well. Subsequently, add the liquid contained in the tube to the drop of blood and consequently, to the same well of the test, using the same pipette. **IT IS CRUCIAL THAT YOU BE CAREFUL and follow each and every one of the mentioned steps.** If necessary, watch the video as many times as you need or contact us; only one kit per participant will be sent.

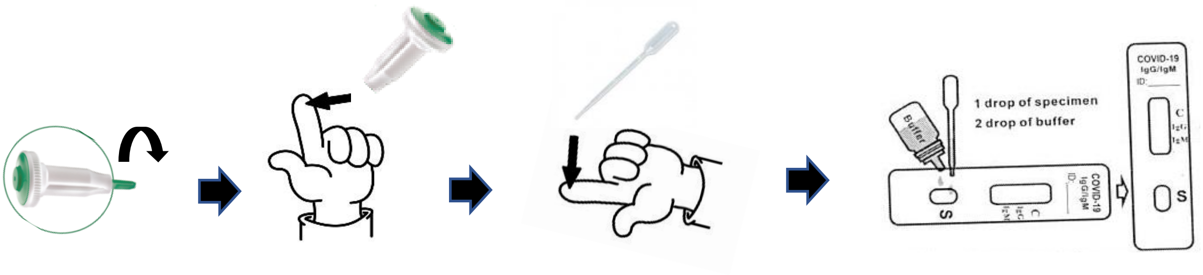

3º) Once you have watched the video and understood the procedure, on the next screen you will find the survey with the questions that you should answer. First of all, do not forget to write both, the Record ID and the kit number, in the corresponding fields. Importantly, you can find these data at the top right of the first page of this document. Then, answer the questions clicking on "continue" to move forward.

4º) Take a picture of the test immediately after putting in the test well both, the drop of blood and the liquid contained in the tube, and, please, attached it clicking the button [[↑] choose file]. Finally, click on "accept".

5º) The test takes 20 minutes to reveal the results. During this time, you can answer the remaining questions by selecting the corresponding option in the drop-down list or by completing them with numbers. You should click on "continue" at the bottom of each page.

6º) Once the 20 minutes have elapse, you should take a second picture of the test indicating the final result. Please, be sure to take a high-quality picture with enough natural light. You can upload the picture just taken to the online application, using your smartphone or computer, or failing that, you can directly connect the camera of your smartphone and take the picture. In case of choosing the last option, the image will be automatically uploaded.

7º) Once the survey is finished and the picture has been correctly uploaded, please, click on "send".

Thank you very much for participate on this study

If you need assistance, please, do not hesitate to contact us calling the phone (+34) [redacted] from Monday to Friday from 9:00 - 17:00h
